# Acetylation at K108 of the NS1 protein is important for the replication and virulence of influenza virus

**DOI:** 10.1186/s13567-020-00747-3

**Published:** 2020-02-24

**Authors:** Jingjiao Ma, Rujuan Wu, Guanlong Xu, Yuqiang Cheng, Zhaofei Wang, Heng’an Wang, Yaxian Yan, Jinxiang Li, Jianhe Sun

**Affiliations:** 1 Shanghai Key Laboratory of Veterinary Biotechnology, Key Laboratory of Urban Agriculture (South), Ministry of Agriculture, School of Agriculture and Biology, Shanghai Jiao Tong University, Shanghai, 200240 China; 2 Chengdu National Agricultural Science and Technology Center, Sichuan, China; 3 China Institute of Veterinary Drug Control, Beijing, 100081 China; 4 College of Veterinary Medicine, Hunan Agricultural University, Changsha, 410128 China

## Abstract

Non-structural protein 1 (NS1) of influenza virus is a multifunctional protein that plays an important role in virus replication and virulence. In this study, an acetylation modification was identified at the K108 residue of the NS1 protein of H1N1 influenza virus. To further explore the function of the K108 acetylation modification of the NS1 protein, a deacetylation-mimic mutation (K108R) and a constant acetylation-mimic mutation (K108Q) were introduced into the NS1 protein in the background of A/WSN/1933 H1N1 (WSN), resulting in two mutant viruses (WSN-NS1-108R and WSN-NS1-108Q). In vitro and mouse studies showed that the deacetylation-mimic mutation K108R in the NS1 protein attenuated the replication and virulence of WSN-NS1-108R, while the constant acetylation-mimic mutant virus WSN-NS1-108Q showed similar replication and pathogenicity as the wild-type WSN virus (WSN-wt). The results indicated that acetylation at K108 of the NS1 protein has an important role in the replication and virulence of influenza virus. To further explore the potential mechanism, the type I interferon (IFN-I) antagonistic activity of the three NS1 proteins (NS1-108Q, NS1-108R, and NS1-wt) was compared in cells, which showed that the K108R mutation significantly attenuated the IFN-β antagonistic activity of the NS1 protein compared with NS1-wt and NS1-108Q. Both NS1-wt and NS1-108Q inhibited the IFN-β response activated by RIG-I CARD domain, MAVS, TBK1, and IRF3 more efficiently than the NS1-108R protein in cells. Taken together, the results indicated that acetylation at NS1 K108 is important for the IFN antagonistic activity of the NS1 protein and virulence of the influenza virus.

## Introduction

Influenza virus non-structural protein 1 (NS1) is a multifunctional protein that is responsible for interacting with cellular factors to antagonize the host antiviral response during viral infection [1]. The major role of the NS1 protein is inhibition of both interferon (IFN) and IFN-stimulated proteins by different mechanisms. NS1 inhibits the transcription of type I IFN by binding the 5′ triphosphate viral double-stranded RNAs generated during viral replication to prevent the recognition of viral genomic material by host Pattern recognition receptors (PRRs), including RIG-I, dsRNA-dependent protein kinase R (PKR), and 2′5′-oligoadenylate synthetase (OAS)/RNase L [2–4]. NS1 can also interact directly with RIG-I in the absence of RNA binding to inhibit the conformational change of RIG-I required for MAVS activation [5]. Moreover, NS1 is able to interrupt mRNA maturation by inhibiting the nuclear export of host mRNAs by binding to host poly(A)-binding protein II (PABPII) and cleavage and polyadenylation specific factor 30 (CPSF30), which are required for host mRNA processing, resulting in the accumulation of IFN pre-mRNAs in the nucleus of infected cells [6]. In addition, NS1 also antagonizes the IFN signalling response by regulating other host factors, such as phosphoinositide 3-kinase (PI3K) activity, Crk-like protein (CRKL), and the JAK-STAT signalling pathway [7–11].

The NS1 protein, typically 202–237 aa in length depending on the strain, contains four functional regions: an RNA binding domain (RBD, 1–73 aa), linker region (LR, 74–88 aa), effector domain (ED, 89–202 aa), and C-terminal “tail” (CTT, 207–237 aa) [12]. Multiple basic amino acids (e.g., 35R, 38R, 41K, and 46R) in the RBD are important for RNA binding activity and suppressing the activation of PKR [12, 13]. The ED plays an important role by targeting multiple host factors, such as PKR, CPSF30, and p85β (PI3K), to inhibit antiviral responses and enhance viral replication [1]. The residue 187W in the ED domain is important for the dimerization of the NS1 protein, and the W187R substitution impaired NS1 dimerization and attenuated the virus in vivo [14]. In addition, residues 186E, 189D, and 194V play important roles in the binding of NS1 to cleavage and polyadenylation specificity factor 30 (CPSF30), and mutations in those residues weaken the binding of NS1 to CPSF30 and impair the ability of the NS1 protein to shut off host gene expression [15, 16].

Post-translational modifications, such as phosphorylation, SUMOylation, and acetylation, are important for protein function. Phosphorylation at 49T, 80T, and 215T of the NS1 protein are important for interferon antagonistic activity and replication of human influenza virus [17, 18]. SUMOylation at positions 219 and 221 of NS1 are crucial for host protein expression shutoff and replication of H5N1 influenza virus [19]. Acetylation is an important post-translational modification that occurs in two forms [20]. One is the co/post-translational acetylation at the N^α^-termini of the nascent polypeptide chains [21]. The other form is acetylation of the ε-amino group of lysine, which was first recognized in histones regulating gene translation [22] and later was found in non-histone proteins [23]. The acetylation status is reversible and well balanced by lysine (K) acetyltransferases (KATs) and lysine deacetylases (KDACs), which are tightly regulated to perform many cellular functions [24]. Dysfunction of the acetylation machinery can inhibit protein functions and consequently lead to severe diseases [25, 26]. Acetylation has been found in multiple proteins of influenza viruses. Acetylation was identified in the NP protein of influenza virus, and deacetylation of the NP protein prevented the virus from assembling functional virus particles [27]. A histone-like sequence (histone mimic) was identified in the NS1 protein of influenza A H3N2, which contributes to suppression of the antiviral response [28]. The N-terminal acetylation of PA-X is required for the host shutoff activity of PA-X and for viral polymerase activity [29, 30]. Acetylation of PA has been reported to be crucial for polymerase activity, and deacetylated PA protein restricts IAV RNA transcription and replication. The influenza virus haemagglutinin (HA) has three conserved cysteine residues (551, 559, and 562) at its C terminus serving as acylation sites that are essential for the formation of fusion pores and infectivity [31].

In the present study, an acetylation modification at K108 of the NS1 protein was identified and characterized. The results showed that the deacetylation-mimic mutation K108R in the NS1 protein attenuated the replication and virulence of the virus in vitro and in vivo. IFN-β antagonist assays indicated that the K108R mutation attenuated IFN antagonistic ability compared with the NS1-wt or NS1-108Q (constant acetylation-mimic) proteins. Overall, this study indicated that acetylation at K108 of the NS1 protein plays an important role in the replication and virulence of influenza virus.

## Materials and methods

### Cells and virus

Madin–Darby canine kidney (MDCK) cells were maintained in Eagle’s minimal essential medium (EMEM, HyClone, Grand Island, USA) with 5% foetal bovine serum (FBS, Gibco, Grand Island, USA), l-glutamine (Gibco), and 1% antibiotic (Gibco). Human embryonic kidney (HEK) 293T cells and adenocarcinomic human alveolar basal epithelial cells (A549 cells) were maintained in Dulbecco’s modified Eagle’s medium (DMEM, HyClone) supplemented with 10% FBS (Gibco), l-glutamine (Gibco), and 1% antibiotic (Gibco). The virus strain A/WSN/1933 H1N1 (WSN), a mouse-adapted human influenza virus, was propagated and titrated in MDCK cells.

### Acetylation screening with mass spectrometry

To identify the putative acetylation sites in influenza viral proteins, mass spectrometry was conducted with concentrated influenza virus. Briefly, the H1N1 virus was propagated in MDCK cells, and then a total of 100 mL of virus stock was prepared. To concentrate the virus, the collected virus was pelleted by centrifugation at 2000 *g* for 10 min to remove the cell debris. Clarified virus supernatants were layered on a 30% (w/v) sucrose cushion and centrifuged at 200 000 *g* for 3 h. The virus pellet was suspended in water and subjected to mass spectrometry analysis performed by PTM Biolabs LLC (Hangzhou, China).

### Construction of plasmids and mutant viruses

To generate mutant viruses, the site mutations K108R (deacetylation-mimic mutation) and K108Q (constant acetylation-mimic) were introduced into the reverse genetic plasmid pHW2000-WSN-NS by a commercial site-directed mutagenesis kit (Invitrogen, Grand Island, USA). The mutant viruses were rescued in the background of WSN-H1N1 virus as described previously [32], resulting in WSN-NS1-108R and WSN-NS1-108Q viruses, and all the mutant viruses were verified by sequencing. Then, the NS1-wt, NS1-108R, and NS1-108Q genes were amplified and cloned into the pcDNA3.0-FLAG expression vector (FLAG-NS1-wt, FLAG-NS1-108R, and FLAG-NS1-108Q).

### Growth kinetic study and Western blotting

The three viruses were inoculated on monolayer MDCK and A549 cells cultured in 12-well plates with multiplicities of infection (MOIs) of 0.001 and 0.01 for each virus, respectively. Each time point was set up in triplicate, and then the samples were collected at 12, 24, 36, and 48 hours post-inoculation (hpi). The supernatants were titrated on MDCK cells cultured in 96-well plates following the Reed and Muench method to calculate TCID_50_/mL. The cells were collected and subjected to Western blotting. Briefly, the cell lysates were separated on a sodium dodecyl sulfate (SDS)-polyacrylamide gel and transferred to a PVDF membrane. The membrane was blocked in PBS containing 5% skim milk and then incubated with rabbit anti-NS1 polyclonal antibody (GenScript, Piscataway, USA) and then with horseradish peroxidase-conjugated secondary anti-mouse antibody (Thermo Fisher, Grand Island, USA). The proteins were visualized by using an ECL kit (Yeasen, Shanghai, China). Additionally, to determine the protein expression levels of the three NS1 expression plasmids, the FLAG-NS1-wt, FLAG-NS1-108Q, and FLAG-NS1-108R plasmids were transfected into 293T cells. Forty-eight hours post-transfection, the cells were collected and subjected to Western blotting.

### Animal study

Forty-eight 4-week-old female BALB/c mice were randomly allocated into four groups, and each group contained 12 mice. Three groups were challenged with the indicated viruses, and one group was challenged with PBS as a control. The mice were inoculated with virus intranasally with 10^5.5^ TCID_50_ of virus in 50 µL solution under slight anaesthesia with CO_2_. The mice were monitored for body weight, clinical signs, and survival rate each day until 14 days post-infection (dpi), and they were euthanized if they lost more than 25% of their original body weight. Three mice from each group were euthanized at 3 and 5 dpi. The mouse lungs were collected for viral titration and cytokine analysis. To quantify the cytokine levels of IL-1β, IFN-β, and TNF-α in mouse lungs, total RNA was extracted from lung tissues, reverse-transcribed and subjected to quantitative real-time polymerase chain reaction as described previously [33].

### Dual-luciferase reporter assay

To detect the IFN-β antagonistic ability of NS1 proteins, 293T cells were transfected with the indicated NS1 expression plasmids (0.2 μg/well) together with a plasmid expressing firefly luciferase under the control of the IFN-β promoter (pGL-IFN-β-LUC, 0.2 μg/well), the Renilla luciferase expressing plasmid pRL-TK (0.07 μg/well), and the IFN-β stimulator poly(I:C) (0.2 μg/well) or a plasmid expressing the active caspase recruitment domain (CARD) of RIG-I (pcDNA-RIG-I 0.2 μg/well), pcDNA-MAVS (0.2 μg/well), pcDNA-TBK1 (0.2 μg/well) or pcDNA-IRF3 (0.2 μg/well) as described previously [34]. Twenty-four hours post-transfection, the cells were lysed and subjected to a dual-luciferase reporter assay kit (Promega, Madison, USA).

### Immunofluorescence assay (IFA)

MDCK cells cultured on glass slides were infected with WSN-NS1-K108R, WSN-NS1-K108Q, or WSN-wt at an MOI of 1. All cells were fixed with 4% paraformaldehyde (PFA) and permeabilized with 0.1% Triton X-100 in PBS at 12, 24, and 36 hpi. To detect the NS1 proteins, the fixed cells were incubated with rabbit anti-NS1 polyclonal antibody (GenScript), followed by FITC-conjugated anti-rabbit IgG antibody (Yeasen), and then the cells were stained with DAPI. All images were obtained on a Leica TCS SP2 confocal microscope (Leica Microsystems Inc., Buffalo Grove, USA).

### Ethics statement and statistical analysis

The animal study was conducted in accordance with the guidelines of the Animal Care and Use Committee of Shanghai Jiao Tong University, and the animal study protocols were approved by Shanghai Jiao Tong University (Approval No. 20181012). All data were analysed using analysis of variance (two-way ANOVA) in GraphPad Prism version 5.0 (GraphPad software Inc., La Jolla, USA); a *P*-value of 0.05 or less was considered significant.

## Results

### Acetylation modification was identified at K108 of the NS1 protein and mutant viruses were generated

**Figure 1:**
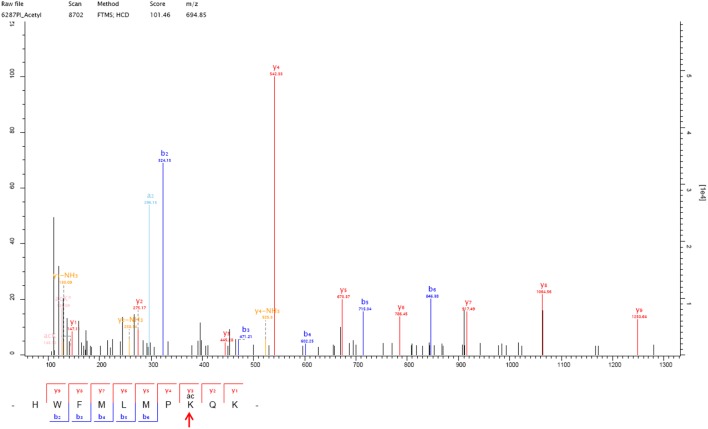
**Mass spectrometry analysis of the acetylation modification at K108 of the NS1 protein of WSN-wt.** One acetylation modification was found at one y3 peak on lysine, which is in the amino acid sequence HWFMLMPKQK of the WSN NS1 protein. The MS/MS spectra obtained with HCD of the + 3 m/z ion at 1021.8967 are shown.

The mass spectrometry results showed that one acetylation modification was identified at position K108 of the NS1 protein of the WSN-wt virus (Figure 1). To further explore whether K108 is subtype-specific, we compared the NS1 amino acid sequences of 1000 randomly selected influenza virus strains of each endemic subtype in birds and humans from GenBank. Most avian H9N2 (100%), H5N1 (99.9%), H7N9 (100%), human H3N2 (99.4%), and human H1N1 (99.9%, isolated before 2009) viruses contained K108 in the NS1 genes, whereas 99.9% of 2009 pandemic H1N1 viruses contained 108R in the NS1 protein. These data demonstrated that the K108 residue is relatively conserved in influenza viruses except the 2009 pandemic H1N1. Since the NS1 protein is an important virulence marker and antagonist of host innate immunity, we chose to further explore the influence of acetylated K108 on virus replication and virulence in this study. To mimic deacetylated lysine, a K108R substitution was introduced into the NS1 protein, since an R substitution prevents acetylation but preserves the positive charge, and a mutant virus containing the NS1-K108R substitution was generated in the background of WSN virus (WSN-NS1-108R). Moreover, to mimic constantly acetylated lysine at K108 of the NS1 protein, a K108Q substitution that is a known acetylation mimic was introduced into NS1, resulting in a mutant virus containing NS1-K108Q (WSN-NS1-108Q).

### Acetylated K108 is important for viral replication in cells

**Figure 2:**
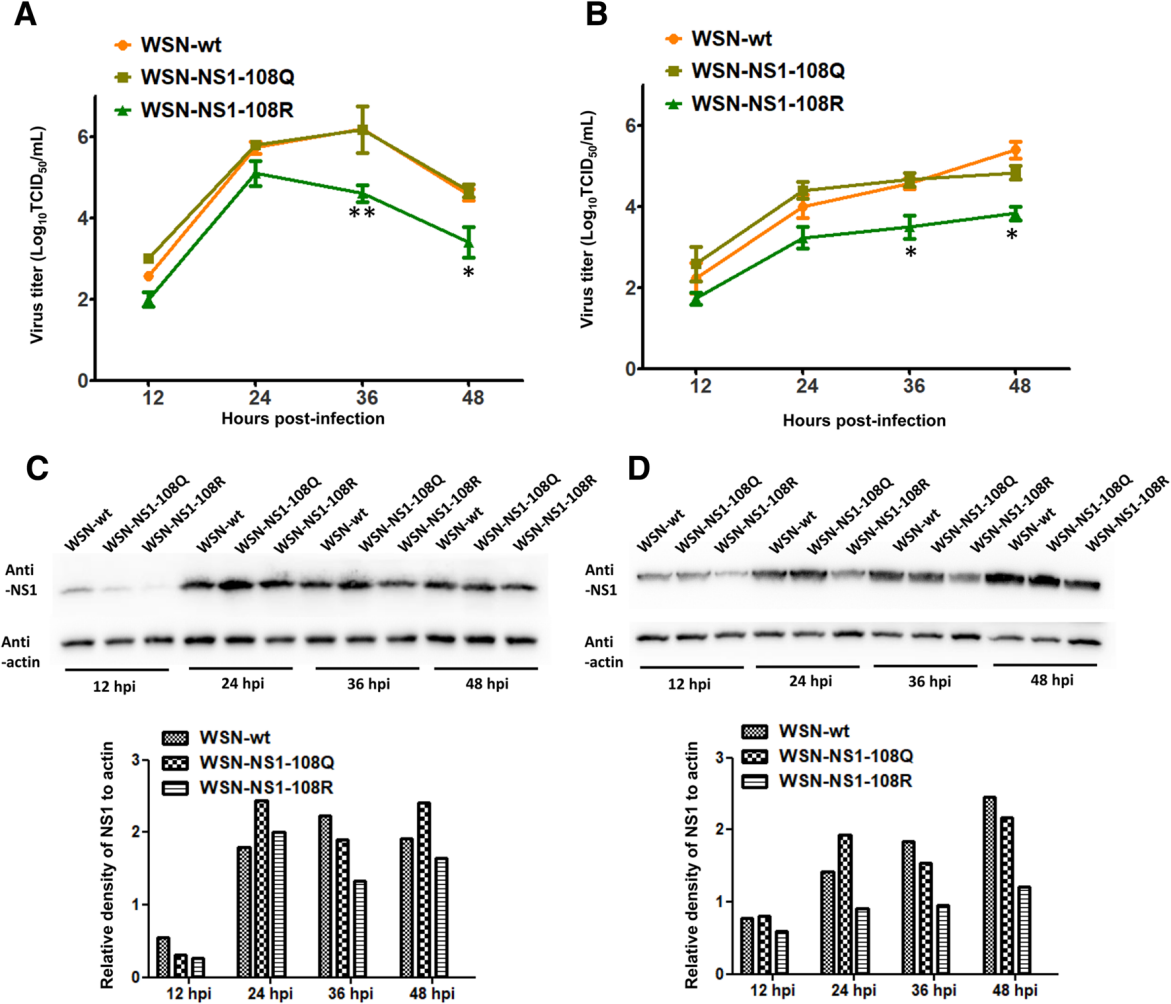
**Growth kinetics and NS1 expression levels of WSN-wt, WSN-NS1-108R, and WSN-NS1-108Q in MDCK and A549 cells.** Monolayers of MDCK (**A**) and A549 (**B**) cells were infected with different MOIs of each virus, and the supernatant samples were collected at the indicated time points. The virus titers were measured in MDCK cells. Each data point indicates the mean ± SEM of three independent experiments, ***P < 0.001, *P < 0.05. Western blotting was conducted to measure the NS1 expression levels of the three viruses in MDCK (**C**) and A549 (**D**) cells, and the relative quantification of NS1 was determined by ImageJ software (version 1.49).

To determine the effect of acetylated K108 on virus replication, MDCK and A549 cells were infected with WSN-wt or the two mutant virus WSN-NS1-108R (deacetylation mimic) or WSN-NS1-108Q (constant acetylation mimic) at the indicated MOIs to obtain multicycle growth curves. All three viruses replicated efficiently in MDCK and A549 cells, and WSN-NS1-108Q and WSN-wt replicated to similar levels at each time point. However, the growth of the deacetylated mutant virus WSN-NS1-108R was significantly impaired compared with that of the other two viruses in MDCK and A549 cells at 36 and 48 hpi, which indicated that the acetylated K108 of NS1 is important for virus replication in vitro at the late stage of infection (Figures 2A and B). Similarly, NS1 protein levels were detected by Western blotting. The results showed that the NS1 expression levels of WSN-NS1-108R were lower than those of WSN-wt and WSN-NS1-108Q at different time points in MDCK and A549 cells (Figures 2C and D).

### Deacetylation-mimic K108R substitution attenuates the mutant virus in a mouse model

**Figure 3:**
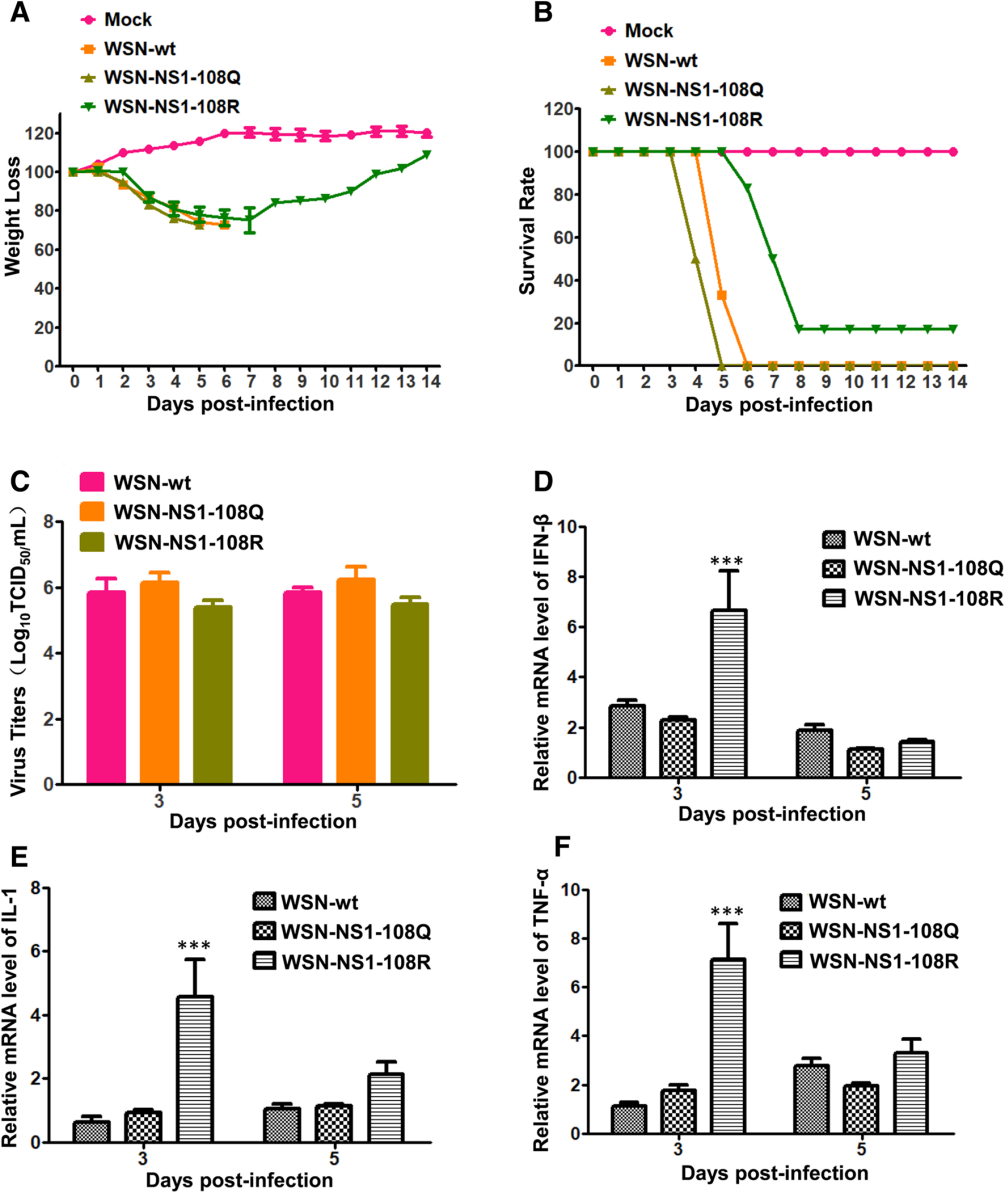
**Pathogenicity of WSN-wt, WSN-NS1-108R, and WSN-NS1-108Q and cytokine production in mice.** Survival rate (**A**) and weight loss (**B**) of infected and mock mice; replication of three influenza viruses in mouse lungs (**C**); relative mRNA levels of IFN-β (**D**), IL-1β (**E**), and TNF-α (**F**) in the infected mouse lungs.

All the mice infected with viruses showed clinical signs such as ruffled fur, depression, and inappetence. The mice infected with constantly acetylated WSN-NS1-108Q displayed more severe clinical signs and started to show mortality earlier than WSN-wt-infected mice. Both WSN-NS1-108Q and WSN-wt caused 100% mortality in infected mice. However, the deacetylated WSN-NS1-108R virus infection resulted in 80% mortality, and the mortality was delayed by 2 days compared with other viruses, which indicated that the deacetylation-mimic K108R substitution attenuated the WSN-NS1-108R virus in mice (Figures 3A and B). Virus titers were slightly lower in the lungs of mice infected with WSN-NS1-108R than in the other two groups (Figure 3C). Notably, significantly higher levels of IFN-β, IL-1β, and TNF-α mRNA were detected in WSN-NS1-108R-infected mice than in the other two groups at 3 dpi but not at 5 dpi (Figures 3D–F), which indicated that WSN-NS1-108R was less efficient at inhibiting the innate immune response than WSN-NS1-108Q and WSN-wt at 3 dpi in mice.

### The acetylated K108 residue is important for the IFN-β antagonistic ability of the NS1 protein

**Figure 4:**
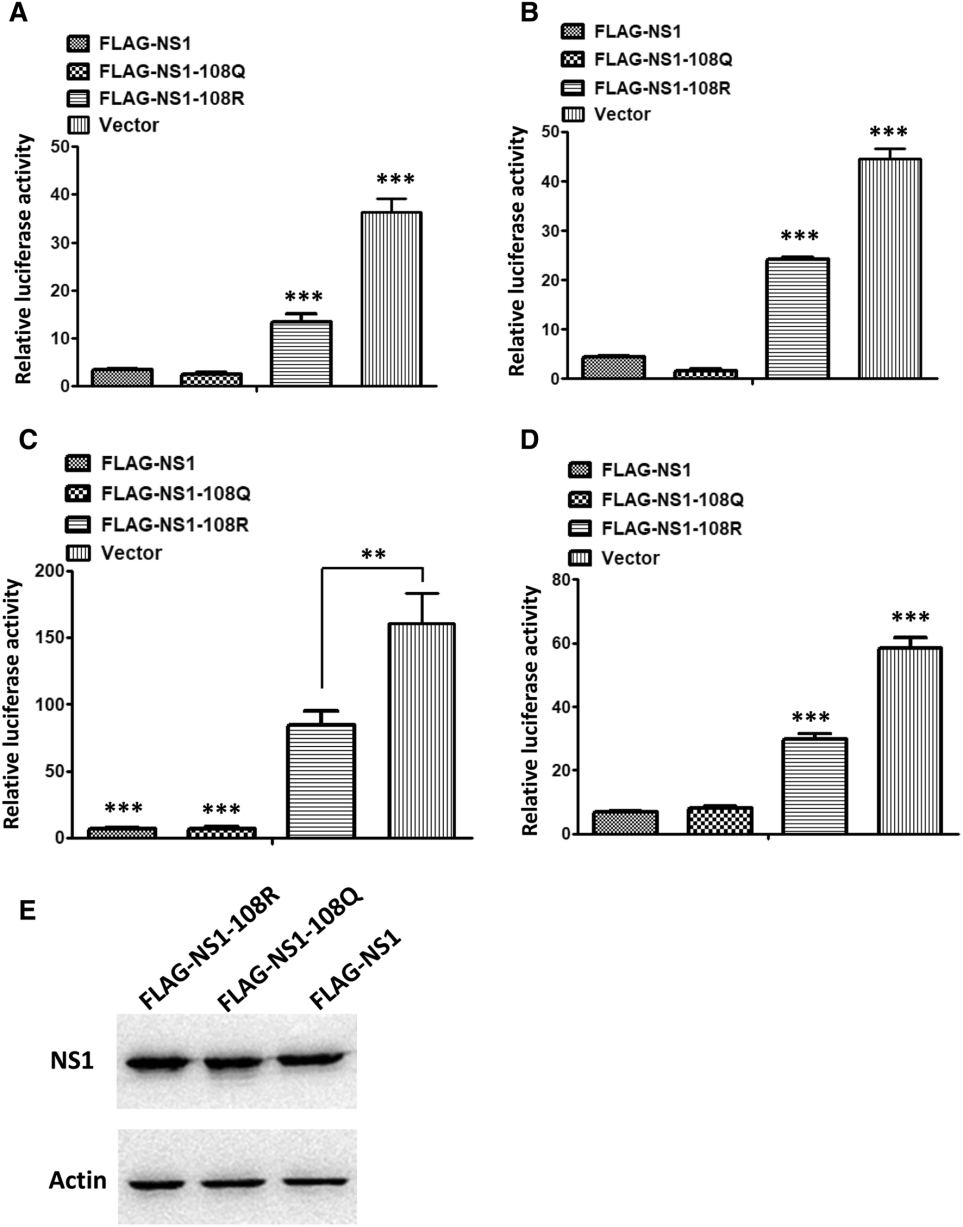
**IFN-β antagonism assay.** IFN-β antagonistic activity of NS1-wt, NS1-108R and NS1-108Q proteins stimulated by Poly(I:C) (**A**), pcDNA-RIG-I (**B**), pcDNA-TBK-1 (**C**), or IRF3 (**D**). Each data point indicates the mean ± SEM of three independent experiments, ***P < 0.001, *P < 0.05.

To determine whether the K108R and K108Q mutations affect the expression of the NS1 protein, the expression levels of FLAG-NS1-wt, FLAG-NS1-108Q, and FLAG-NS1-108R in 293T cells were compared. The three proteins were expressed at similar levels in transfected 293T cells, which indicated that the K108R and K108Q mutations did not influence protein expression (Figure 4E).

The major function of the NS1 protein is inhibition of type I IFN induction, and the acetylated K108 residue is located in the effector domain of NS1, which is important for its IFN antagonistic ability. To determine the effect of the acetylated K108 residue on IFN suppression by the NS1 protein, the inhibition of IFN-β promoter activity by FLAG-NS1-wt, FLAG-NS1-108Q, and FLAG-NS1-108R was evaluated. The results showed that FLAG-NS1-wt and acetylation-mimic FLAG-NS1-108Q suppressed the IFN-β promoter activity stimulated by poly(I:C) (Figure 4A); however, the deacetylation-mimic FLAG-NS1-108R protein was significantly less capable of inhibiting the activation of the IFN-β promoter compared with the acetylated NS1 proteins (Figure 4A). This result indicated that the impaired IFN-β antagonistic ability might be responsible for the attenuation of the WSN-NS1-108Q virus in vitro and in vivo.

Influenza virus infection stimulates type I IFN production by signal transduction from RIG-I to TBK1 to IRF3. To further explore how the K108R mutation attenuated the IFN-β antagonistic ability of the NS1 protein, we co-transfected NS1 expression plasmids, an IFN-β reporter plasmid and different type I interferon pathway components, including RIG-I CARD, TBK1, and the active form of IRF3, into 293T cells. The results showed that NS1-108Q and NS1-wt inhibited the IFN-β response stimulated by each component more efficiently than NS1-108R, which suggested that the acetylated K108 residue is important for inhibiting the IFN-β response of NS1 that targets factors downstream of IRF3 or other proteins (Figures 4B–D).

### Deacetylation-mimic K108R substitution retains the NS1 protein in the cytoplasm of infected cells

**Figure 5:**
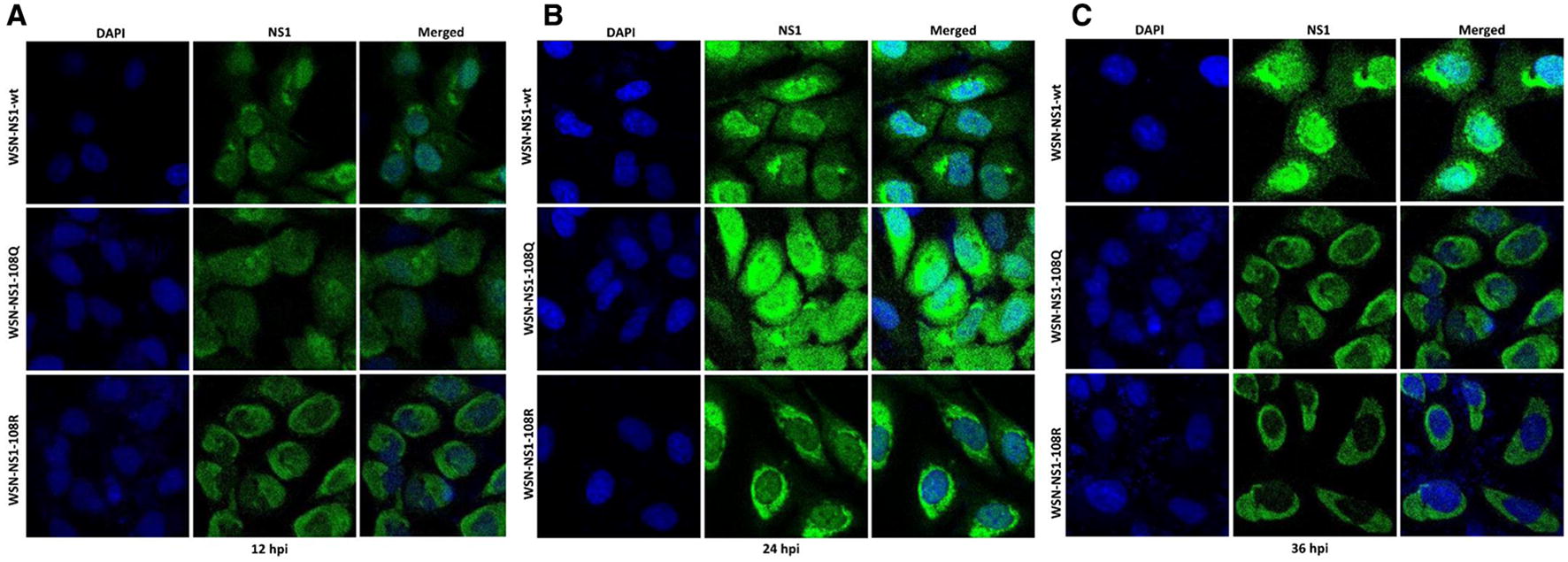
**Localization of NS1 proteins in infected MDCK cells.** The MDCK cells were infected with WSN-NS1-108R, WSN-NS1-108Q, or WSN-wt. The cells were fixed, permeabilized at 12 (**A**), 24 (**B**), and 36 (**C**) hours post-infection and probed with an anti-NS1 antibody, followed by DAPI staining. NS1 (green) and nuclei (blue) were visualized by confocal microscopy.

Two nuclear localization signals (35–41 and 216–221) have been identified in the NS1 protein, which drive NS1 to the nucleus during the early stage of infection. To determine whether K108R changes the subcellular localization of NS1 during infection, MDCK cells were infected with the three viruses. The NS1 protein of WSN-wt was located in the nucleus and cytoplasm of infected cells at 12 and 24 hpi (Figures 5A and B). At the late stage of infection (36 hpi), the NS1 protein was mainly located in the nucleus and perinuclear area of infected cells (Figure 5C). The NS1-108Q protein was located in both the nucleus and cytoplasm at 12 hpi and 24 hpi, while it was mainly located in the perinuclear area of infected cells at 36 hpi. In contrast, the NS1-108R protein accumulated mostly in the cytoplasm during the whole infection course. This result indicated that the deacetylation-mimic K108R substitution retained NS1 protein in the cytoplasm of infected cells, suggesting that the acetylated K108 residue is important for the nuclear localization of the NS1 protein (Figures 5A–C).

## Discussion

Post-translational modification is important for protein function, stability, cellular localization, and protein–protein interactions. Recent studies have shown that post-translational modifications of viral proteins modulate the virus life cycle, e.g., phosphorylation of influenza viral proteins (NS1, M1, and NP) plays important roles in virus replication [35–38]. The ubiquitination of NP and M2 proteins is crucial for viral RNA replication and the production of infectious virus particles [37]. Acetylation is an important post-translational modification in eukaryotes, but the occurrence and function of acetylation in influenza viral proteins remain largely unclear. Giese et al. reported that acetylation of 77 K, 113 K, and 229 K of NP proteins is important for virus polymerase activity and replication [27]. In the present study, we identified and characterized the acetylation of K108 in the NS1 protein, and the deacetylation of K108Q affected viral replication in cells at 36 and 48 hpi. The expression levels of NS1-K108Q and NS1-K108R in transfected 293T cells were similar (Figure 4E), while the NS1 levels in infected MDCK and A549 cells were different (Figures 2C and D). This result could be attributed to the deacetylation affecting virus replication, which resulted in low expression of NS1 in infected cells. Moreover, the acetylation of 108Q contributes to the IFN antagonistic ability of the NS1 protein. The mRNA levels of IFN-β, IL-1β, and TNF-α in mouse lungs of the WSN-NS1-K108R-infected group were significantly higher than those in the other two groups at 3 dpi, which indicated that NS1-108R was less efficient at inhibiting the production of innate antiviral cytokines at 3 dpi in mice.

The NS1 protein of influenza virus is a virulence factor that inhibits the antiviral immunity of the infected host, and C-terminal truncation has been widely used as a strategy to generate attenuated virus vaccine candidates [39]. One mechanism used by NS1 to inhibit the IFN response is through direct binding and sequestration of RNA as well as direct interaction with TRIM25 and complex formation with the RNA sensor RIG-I, resulting in inhibition of the activation of the RIG-I CARD and hence inhibition of IRF3 activation [12]. The RNA binding, RIG-I and TRIM25 interacting domains are located in the N-terminus (1–73 aa) of the NS1 protein. However, the acetylated K108 is located in the ED domain, and acetylation of K108 may not affect the RNA binding capability of the NS1 protein. The NS1-K108R substitution impaired the suppression of IFN promoter activation by poly(I:C), RIG-1 CARD, TBK-1, and IRF3, which suggested that the NS1-K108R substitution affected the IFN antagonism of NS1 either through targeting downstream of IRF3 or a general mechanism that NS1 uses to inhibit IFN, such as interaction with CPSF30, resulting in inhibition of the processing of mRNA, including IFN mRNA [6]. Notably, the CPSF30 protein is mainly located in the nucleus and is required for the 3′ end processing of all host pre-mRNAs. Interestingly, the deacetylation-mimic K108R substitution retained NS1 protein in the cytoplasm of infected cells, resulting in a possible impaired interaction between CPSF30 and the NS1 protein, subsequently leading to attenuated IFN antagonism. Benjamin Hale and colleagues found that the A/California/04/2009 (H1N1) virus has 108R in NS1, and NS1 was unable to suppress general host gene expression. Nevertheless, the RK108 substitution in the NS1/2009 protein restored its ability to block general gene expression and bind CPSF30 [40]. This could explain why attenuation of the IFN antagonistic ability of NS1-K108R is independent of RIG-I CARD, TBK-1, and IRF3 activation. In addition, Anastasina et al. [41] reported that the NS1 protein binds to cellular DNA to block the cellular transcription of IFNs and ISGs; thus, NS1 proteins retained in the cytoplasm lose their cellular DNA binding function, resulting in impaired IFN-β antagonistic ability.

Two known nuclear localization sequences (NLSs) of NS1 proteins are located at the 35–41 and 219–232 positions. The 35–41 NLS of the NS1 protein is highly conserved among influenza A virus strains [42]. The second NLS (219–232) is virus strain specific, and the 2009 pandemic H1N1 lacks the second NLS in the NS1 protein [43]. Single point mutations, either R35A, R38A, or K41A, completely eliminated importin protein binding, which transports target proteins to the nucleus [42]. Notably, in this study, acetylation of K108 located outside of the NLS affected the cellular localization of NS1 protein, and the deacetylation-mimic K108R substitution blocked the nuclear localization of the NS1 protein in infected cells; however, the underlying mechanism remains unknown.

Interestingly, the NS1-K108 residue is relatively conserved in most influenza viruses, except for the 2009 pandemic H1N1. The 2009 pandemic H1N1 has 108R in NS1, which causes inefficient general host gene expression shutoff, while R108K restores its ability to block general host genes and bind CPSF30 [40]. Potentially, the 2009 pandemic H1N1 virus might use different strategies to overcome the IFN response compared with the other influenza viruses. Overall, we identified an acetylation of K108 of the NS1 protein of influenza virus, and the acetylation of K108 plays an important role in the cellular localization, IFN antagonistic ability, replication, and virulence of influenza virus.
